# Ib期上叶肺癌纵隔淋巴结清扫方式的研究

**DOI:** 10.3779/j.issn.1009-3419.2013.11.04

**Published:** 2013-11-20

**Authors:** 文利 王, 峰 毛, 屠阳 申, 运清 梅

**Affiliations:** 1 200065 上海，同济大学附属同济医院心胸外科 Department of Thoracic and Cardiovascular Surgery, Tongji Hospital, Affliated to Tongji University, Shanghai 200065, China; 2 200030 上海，上海交通大学附属胸科医院/上海市肺部肿瘤临床医学中心胸外科 Department of Thoracic Surgery, Shanghai Chest Hospital/Shanghai Lung Tumor Clinical Medical Center, Shanghai 200030, China

**Keywords:** 肺肿瘤, Ib期, 淋巴结清扫, 预后, Lung neoplasms, Stage Ib, Lymphadenectomy, Prognosis

## Abstract

**背景与目的:**

淋巴转移是肺癌最主要的转移途径，也是影响肺癌患者预后的主要因素之一。现有的研究显示上叶肺癌较之中、下叶肺癌更易发生区域性纵隔淋巴结转移。本研究回顾分析Ib期上叶非小细胞肺癌（non-small cell lung cancer, NSCLC）纵隔淋巴结清扫方式的选择及影响预后的相关因素。

**方法:**

147例行肺上叶完全性切除术的NSCLC患者，其中左肺上叶71例，右肺上叶76例。术后病理均为Ib期（T2aN0M0）。术中共清扫淋巴结925枚，其中纵隔淋巴结491枚（上纵隔组266枚，下纵隔组225枚）。采用*Kaplan-Meier*乘积法和*Log-rank*检验对患者进行单因素生存分析，采用*Cox*回归模型进行多因素生存分析。

**结果:**

① 单因素及多因素分析均显示：年龄、肿瘤直径及上纵隔淋巴结清扫站数是影响患者预后的重要因素；②对于Ib期右肺上叶NSCLC，#4组淋巴结与预后存在统计学意义（*P*=0.021），而对于Ib期左肺上叶NSCLC，#5组淋巴结与预后存在统计学意义（*P*=0.024）。

**结论:**

对于Ib期上叶NSCLC而言，年龄、肿瘤直径及上纵隔淋巴结清扫站数是影响患者预后的重要因素；对于此类患者，采用肺叶特异性系统性淋巴结清扫或许是更为高效的手术方式。

## Abstract (en)

**Background and objective:**

Lymphatic metastasis is the most important way for the spread of lung cancer and is one of the important factors affecting the prognosis. Existing studies showed that compared to middle or lower lobe NSCLC, upper lobe non-small cell lung cancer (NSCLC) has a higher probability of occurring regional mediastinal lymph node metastasis. The purpose of this study is to research the prognostic factors and lymphadenectomy of stage Ib upper lobe NSCLC.

**Methods:**

A retrospective study of 147 consecutive subjects (76 and 71 for right and left upper lobe NSCLC respectively) who had undergone curative resection for stage Ib upper lobe NSCLC was performed. A total of 925 lymph nodes were removed during the surgery in all enrolled patients and a total of 491 mediastinal lymph nodes (266 and 225 for superior and inferior mediastinal lymph nodes respectively) were removed. *Kaplan-Meier* product method and *Log-rank* test were used for univariate survival analysis and *Cox* regression model was used for multivariate survival analysis.

**Results:**

① Both univariate and multivariate analysis showed that age, tumor size and number of removed superior mediastinal lymph node stations were the important prognostic factors of stage Ib upper lobe NSCLC; ②For stage Ib right upper lobe NSCLC, station 4 lymph node was of statistical significance to the prognosis (*P*=0.021); while for stage Ib left upper lobe NSCLC, station 5 lymph node was of statistical significance to the prognosis (*P*=0.024).

**Conclusion:**

In surgically treated stage Ib upper lobe NSCLC patients, age, tumor size and number of removed superior mediastinal lymph node stations are the important prognostic factors. And in this kind of patients, lobe-specific systematic lymph node dissection may be a more efficient procedure during the surgery.

淋巴转移是肺癌最主要的转移扩散途径，也是影响预后的重要因素之一。现有研究显示，肺癌淋巴结转移的方式按淋巴结引流途径存在一定的规律，即按照肺内淋巴结→肺门淋巴结→纵隔淋巴结的顺序发生转移。与中、下叶非小细胞肺癌（non-small cell lung cancer, NSCLC）相比，上叶NSCLC更易发生纵隔淋巴结转移，且其转移多局限于上纵隔区域淋巴结，而中、下叶NSCLC则易出现上、下纵隔跳跃式淋巴结转移^[1, 2]^。本文回顾性分析147例完全性切除的Ib期上叶NSCLC患者，探讨纵隔淋巴结的适宜清扫方式，同时研究影响患者预后的相关因素。

## 1 资料与方法

### 1.1 病例选择及纳入标准

选择2001年5月-2004年12月于上海胸科医院行完全性切除术的147例Ib期上叶NSCLC患者。入组标准：①原发病灶位于左肺/右肺上叶；②术前除一般常规检查外，均行增强胸部CT及头颅CT扫描、腹部B超、全身骨同位素扫描以排除远处转移；③手术为完全性切除，参照2005年IASLC提出的肺癌完全性切除手术标准^[3]^；④术后病理诊断为NSCLC，病理分期按第7版肺癌TNM分期重新分期，为T2aN0M0（Ib期）。剔除新辅助化疗、术后辅助化疗病例及非肿瘤原因死亡病例。

### 1.2 资料收集

对所有入组病例采集以下数据：住院号、手术日期、性别、年龄、肿瘤直径、肿瘤部位、病理类型、肿瘤分化程度、脏层胸膜侵犯情况、淋巴结清扫数、纵隔淋巴结清扫数及上纵隔淋巴结清扫站数。

### 1.3 随访

147例Ib期上叶NSCLC患者的术后随访生存期按月计算，死亡患者以手术日距死亡日的差值计算，生存患者以手术日距末次随访日的差值计算。随访数据来源于上海市疾病控制中心，随访截止日期为2009年12月30日。

### 1.4 统计学分析

采用SPSS 15.0统计软件包进行数据整理及统计分析。生存分析采用*Kaplan-Meier*乘积法和*Log-rank*检验，多因素分析采用*Cox*回归模型。*P* < 0.05为差异具有统计学意义。

## 2 结果

### 2.1 入组患者资料

符合入组标准的患者共147例，患者临床特征情况如表 1所示。其中右肺上叶组76例，左肺上叶组71例。两组年龄、性别、病理类型、肿瘤分化程度、脏层胸膜侵犯及肿瘤直径无统计学差异（*P* > 0.05）。入组患者共清扫淋巴结925枚（每例平均6.29枚），其中纵隔淋巴结共491枚，清扫上纵隔淋巴结 < 2站者55例，≥2站者92例。两组间淋巴结清扫数及纵隔淋巴结清扫数无统计学差异（*P* > 0.05）。而右肺上叶组清扫上纵隔淋巴结站数明显多于左肺上叶组（*P*=0.004），此项差异主要是由于左右上纵隔固有的解剖因素所致。

**1:** 147例入组患者临床特征 Characteristics of 147 patients with stage Ib upper lobe non-small cell lung cancer

Characteristics	*n* (%)	Right upper lobe [*n* (%)]		Left upper lobe [*n* (%)]	*P*
Age (yr)					0.30
< 58	51 (34.7)	30 (39.5)		21 (29.6)	
58-68	56 (38.1)	29 (38.2)		27 (38.0)	
> 68	40 (27.2)	17 (22.4)		23 (32.4)	
Sex					0.09
Female	64 (43.5)	28 (36.8)		36 (50.7)	
Male	83 (56.5)	48 (63.2)		35 (49.3)	
Histological type					0.77
Adenocarcinoma	96 (65.3)	50 (65.8)		46 (64.8)	
Squamous	37 (25.2)	20 (26.3)		17 (23.9)	
Adenosquamous	14 (9.5)	6 (7.9)		8 (11.3)	
Grade of differentiation					0.82
Well	74 (50.3)	41 (53.9)		33 (46.5)	
Moderately	53 (36.1)	26 (34.2)		27 (38.0)	
Poorly	13 (8.8)	6 (7.9)		7 (9.9)	
Unknown	7 (4.8)	3 (3.9)		4 (5.6)	
Visceral pleura invasion					0.73
Present	129 (87.8)	66 (86.8)		63 (88.7)	
Absent	18 (12.2)	10 (13.2)		8 (11.3)	
Tumor size (cm)					0.07
≤3	60 (40.8)	37 (48.7)		23 (32.4)	
3.1-5	87 (59.2)	39 (51.3)		48 (67.6)	
Number of removed LN					0.22
< 7	112 (76.2)	52 (68.4)		60 (84.5)	
≥7	35 (23.8)	24 (31.6)		11 (15.5)	
Number of removed MLN					0.06
< 4	106 (72.1)	47 (61.8)		59 (83.1)	
≥4	41 (27.9)	29 (38.2)		12 (16.9)	
Number of removed SMLNS					0.004
< 2	55 (37.4)	20 (26.3)		35 (49.3)	
≥2	92 (62.6)	56 (73.7)		36 (50.7)	
LN: lymph nodes; MLN: mediastinal lymph nodes; SMLNS: superior mediastinal lymph node stations					

### 2.2 总体生存状态

147例Ib期NSCLC患者总体3年及5年生存率分别为76.2%和70.7%；其中右肺上叶组分别为80.2%和73.5%，左肺上叶组分别为71.8%和67.6%。两组间生存率无统计学差异（*P*=0.240）（图 1）。

**1:**
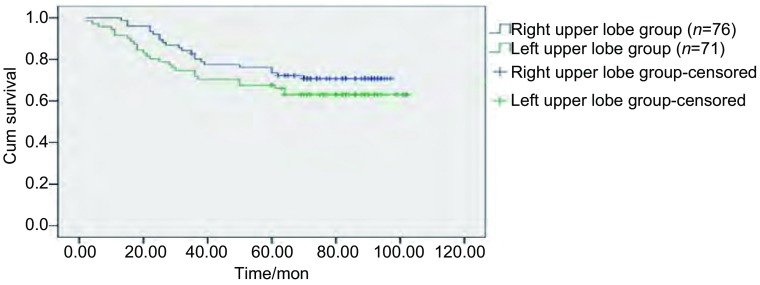
两组间患者*Kaplan-Meier*累积生存时间曲线分析。 *Kaplan-Meier* cumulative survival time curves according to pulmonary location: right upper lobe group *vs* left upper lobe group.

### 2.3 *Cox*回归模型多因素生存分析

将147例患者的年龄、性别、肿瘤部位、肿瘤分化程度、脏层胸膜侵犯、病理类型、肿瘤直径、淋巴结清扫数、纵隔淋巴结清扫数、上纵隔淋巴结清扫站数等变量代入*Cox*回归模型进行多因素生存分析。结果显示：年龄、肿瘤直径及上纵隔淋巴结清扫站数是影响Ib期上叶肺癌生存率的重要预后因素（表 2）。

**2:** 影响Ib期上叶肺癌生存率的预后因素（*Cox*回归分析） Prognostic factors for patients with stage Ib upper lobe NSCLC (*Cox* regression model)

Factors	*P*	Hazard ratio (HR)	95%CI	
			Lower	Upper
Age	0.001	1.075	1.031	1.120
Gender	0.508	0.772	0.359	1.660
Tumor location	0.434	1.283	0.687	2.396
Grade of differentiation	0.058	0.795	0.628	1.008
Visceral pleura invasion	0.074	0.412	0.156	1.091
Histological type	0.927	0.975	0.568	1.673
Tumor size	0.000, 9	1.468	1.212	1.777
Number of removed LN	0.665	0.666	0.105	4.204
Number of removed MLN	0.424	1.871	0.403	8.694
Number of removed SMLNS	0.003	0.446	0.263	0.757

### 2.4 *Kaplan-Meier*单因素生存分析

对147例NSCLC患者进行单因素*Kaplan-Meier*分析，结果显示：年龄 < 58岁组、58岁-68岁组及 > 68岁组之间生存率存在统计学差异（*P*=0.008），各组之间生存率亦存在统计学差异（年龄 < 58岁组*vs*58岁-68岁组，*P*=0.015；58岁-68岁组*vs* > 68岁组，*P*=0.036）；肿瘤直径≤3 cm组、3.1 cm-5 cm之间生存率存在统计学差异（*P*=0.005）；清扫上纵隔淋巴结≥2站者预后明显预后优于 < 2站者（*P*=0.006）。而生存率与性别、脏层胸膜侵犯、病理类型、肿瘤分化程度、淋巴结清扫数、纵隔淋巴结清扫数等无相关性（表 3，图 2）。

**3:** Ib期上叶NSCLC *Kaplan-Meier*单因素分析 Univariate analyses of prognostic factors of stage Ib upper lobe non-small cell lung cancer (NSCLC)

Factors	*n*	%	*χ*^2^	*P*
Age (yr)			18.584	0.008
< 58	51	34.7%		
58-68	56	38.1%		
> 68	40	27.2%		
Sex			1.225	0.268
Female	64	43.5%		
Male	83	56.5%		
Histological type			4.519	0.104
Adenocarcinoma	96	65.3%		
Squamous	37	25.2%		
Adenosquamous	14	9.5%		
Grade of differentiation			13.857	0.067
Well	74	50.3%		
Moderately	53	36.1%		
Poorly	13	8.8%		
Unknown	7	4.8%		
Visceral pleura invasion			0.228	0.633
Present	129	87.8%		
Absent	18	12.2%		
Tumor size (cm)			7.892	0.005
≤3	60	40.8%		
3.1-5	87	59.2%		
Number of removed LN			1.996	0.158
< 7	112	76.2%		
≥7	35	23.8%		
Number of removed MLN			1.663	0.197
< 4	106	72.1%		
≥4	41	27.9%		
Number of removed SMLNS			8.798	0.006
< 2	55	37.4%		
≥2	92	62.6%		

**2:**
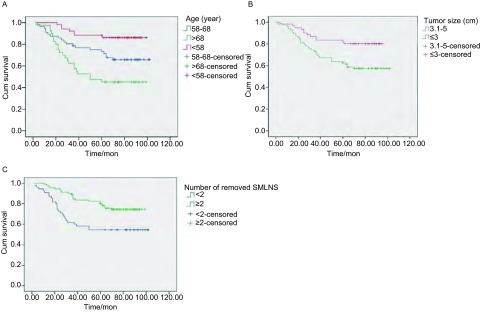
*Kaplan-Meier*累积生存时间曲线分析 *Kaplan-Meier* cumulative survival time curves. A: According to age; B: According to tumor size; C: According to Number of removed SMLNS.

### 2.5 上纵隔淋巴结清扫对预后的影响

采用单因素*Kaplan-Meier*单因素生存分析上纵隔淋巴结清扫对于Ib期上叶NSCLC预后的影响，结果显示：对于右肺上叶NSCLC，常规手术可以清扫范围内的右上纵隔淋巴结（#2，#3，#4组）中，#4组淋巴结对患者预后的影响存在统计学意义（*P*=0.021）；对于左肺上叶NSCLC，常规手术可以清扫范围内的左上纵隔淋巴结（#4L，#5，#6组）中，#5组淋巴结对患者预后的影响存在统计学意义（*P*=0.024）（表 4）。

**4:** 上纵隔淋巴结清扫对Ib期上叶NSCLC预后的分析 Univariate analyses according to removed superior mediastinal lymph node station

Station	Right upper lobe NSCLC			Station	Left upper lobe NSCLC		
	*n* (%)	*χ*^2^	*P*		*n* (%)	*χ*^2^	*P*
#2		0.038	0.845	#4L		0.060	0.983
Removed	36 (47.4)			Removed	19 (26.8)		
Unremoved	40 (52.6)			Unremoved	52 (73.2)		
#3		0.062	0.803	#5		5.349	0.024
Removed	60 (78.9)			Removed	59 (83.1)		
Unremoved	16 (21.1)			Unremoved	12 (16.9)		
#4R		5.329	0.021	#6		0.142	0.706
Removed	53 (69.7)			Removed	29 (40.8)		
Unremoved	23 (30.3)			Unremoved	42 (59.2)		

## 3 讨论

目前，Ib期NSCLC患者的治疗仍以手术为主，基本方式为肺癌（全肺）切除术+淋巴结清扫术，但是淋巴结清扫范围依然存在较大争议，特别是对于上叶NSCLC。虽然研究证实上叶NSCLC发生纵隔淋巴结转移的概率高于中、下叶NSCLC，但是其纵隔淋巴结的转移往往局限于上纵隔区域淋巴结，而中、下叶NSCLC则易出现上、下纵隔跳跃式淋巴结转移^[1, 4, 5]^。因此，根据上叶肺癌转移特点，结合Ib期上叶NSCLC预后因素的研究，有助于早期肺癌适宜淋巴结清扫方式的选择。

年龄因素一直是影响肺癌（特别是早期肺癌）预后的争议性问题。Mery等^[6]^通过控制性别、病理类型、病理分期及手术类型等因素，揭示年龄是影响Ⅰ期-Ⅱ期NSCLC患者生存率的重用预后因素。Agarwal等^[7]^亦证实在Ⅰ期-Ⅱ期NSCLC患者中，死亡率随年龄的增加而呈现急剧升高趋势（年龄每增加1岁，HR将增高近6%），因此认为年龄是生存率的重要预测指标。本研究结果亦提示年龄是影响Ib期上叶NSCLC患者生存率的重要影响因素之一：年龄每增加1岁，其HR将增高近7.5%。但也有学者认为年龄并非影响早期肺癌生存率的重要预后因素^[8]^。特别是对于老年患者而言，单独以年龄因素来评估预后并不科学。老年患者除了要面临肿瘤相关性因素的威胁外，还需面临合并症及脏器功能受损等非肿瘤相关性因素的影响。荷兰一项针对43, 111例癌症患者诊断时合并症发生率的研究发现，65岁以上的癌症患者，诊断时伴有一项严重合并症的为64岁以下的1.4倍，最常见的合并症为心血管疾患。70岁以上的NSCLC术后死亡率（11%）远高于低龄的患者（2%, *P* < 0.01），心血管和血栓性事件也多见于70岁以上的肺癌患者^[9]^。

与年龄因素存在争议不同，肿瘤直径一直被认为是影响肺癌患者预后的重要因素。特别是对于Ⅰ期NSCLC，肿瘤直径对于预后影响的研究一直在进行。Okada等^[10]^通过对1, 465例完全性手术切除的NSCLC患者分析发现，对于Ⅰ期肺癌，病灶大小是影响其预后的独立因素。Christian等^[11]^通过回顾性地研究548例完全性切除的Ⅰ期NSCLC患者报道：肿瘤直径2 cm和5 cm是两个危险阈值，肿瘤直径一旦达到以上阈值，其死亡风险比相应增加58%及118%。2009年第7版肺癌分期标准中，T2被细分为T2a和T2b，直径 > 7 cm者划归为T3，而仅T2aN0M0被列为Ib期，T2bN0M0及T3N0M0则分别为Ⅱa期、Ⅱb期^[12]^，凸显肿瘤直径是衡量NSCLC的独立因素之一。本研究结果显示，对于Ib期上叶NSCLC患者，肿瘤直径是影响其预后的独立因素之一：肿瘤直径每增大1 cm，其HR可上升28.3%；肿瘤直径≤3 cm组、3.1 cm-5 cm组之间生存率存在差异（*P*=0.005）。但也有部分学者认为，不能简单地将肿瘤直径大小作为影响NSCLC预后的独立指标，而应与病理类型相结合。Lin等^[13]^认为对于腺癌而言，应将2.5 cm设为早期NSCLC的临界值，而对于鳞癌则可放宽至4 cm。

近年来，对于Ib期NSCLC患者是否应行系统性淋巴结清扫一直存在着争议。吴一龙等^[14]^通过前瞻性研究报道：对于Ⅰ期NSCLC，系统性淋巴结清扫可更彻底地清除肿瘤细胞，降低术后复发及远处转移的几率。而Izbicki等^[15]^的研究则认为对于处于N0状态的患者，系统性淋巴结清扫并不能提高患者的生存率。Ishiguro等^[16]^通过临床回顾性研究报道，对于临床病理分期为Ⅰ期的NSCLC患者，术中行肺叶特异性系统淋巴结清扫，其5年生存率与系统性淋巴结清扫组相比无统计学意义。本研究的结果提示，淋巴结清扫数及纵隔淋巴结清扫数对于Ib期上叶NSCLC患者的预后无统计学意义（*P* > 0.05）。上纵隔淋巴结清扫站数是影响Ib期上叶NSCLC患者预后的重要因素，且上纵隔淋巴结清扫≥2站者预后明显优于清扫上纵隔淋巴结 < 2站者（*P*=0.006）。此结果可能与上叶肺癌倾向于发生上纵隔区域淋巴结转移，且较少发生非区域淋巴结转移有关。进一步的研究提示，对于右肺而言，术中是否切除#4R组淋巴结是影响预后的重要因素（*P*=0.021）；对于左肺，是否切除#5组淋巴结是影响预后的重要因素（*P*=0.024）。此结果提示：①部分Ib期上叶NSCLC患者上纵隔淋巴结可能已存在跳跃性微转移，但是由于现有病理学检测方法的局限性，无法对其正确地检测。而随着分子生物学技术的发展，目前已有学者提出通过检测基因组或分子标志物，从而进一步提高病理诊断的准确性^[17, 18]^；②根据上叶肺癌转移的特点，#4R组及#5组淋巴结作为右/左肺上叶的前哨淋巴结，发生跳跃性微转移的可能性较大。这或许可以解释为何清扫以上两站淋巴结可改善Ib期右/左肺上叶NSCLC患者的预后。除此之外，Masashi等^[19]^认为由于#3R组及#6组淋巴结亦是转移高发区，故右肺上叶NSCLC在清扫#4组淋巴结的同时，应清扫#3组淋巴结；而左肺上叶NSCLC，清扫#5组淋巴结的同时，应对#6组淋巴结进行清扫。

基于本研究有限样本的研究，对于Ib期上叶NSCLC而言，患者的年龄、肿瘤直径及上纵隔淋巴结清扫站数是影响预后的重要因素，上叶特异性纵隔淋巴结清扫可能是一种更为高效的淋巴结清扫方式，但其范围仍有待进一步研究。
